# Supplementary material for: Anti-Inflammatory CeO2 Nanoparticles Prevented Cytotoxicity Due to Exogenous Nitric Oxide Donors via Induction Rather Than Inhibition of Superoxide/Nitric Oxide in HUVE Cells
Source: Molecules. 2021 Sep 6;26(17):5416. doi: 10.3390/molecules26175416 (PMC8434366; doi:10.3390/molecules26175416)
Supplement: Supplementary file 1 [file molecules-26-05416-s001.zip › molecules-1318315-supplementary.pdf]

Supplementary Materials

S1

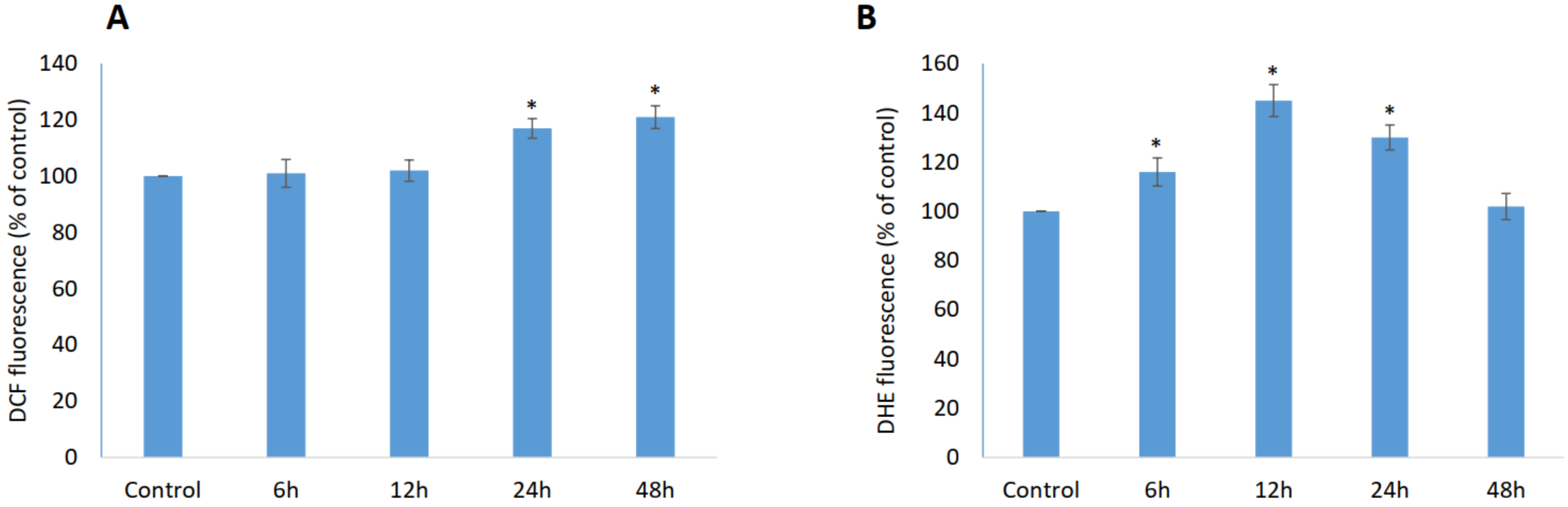

Figure S1: Time-dependent measurement of ROS using DCF- and DHE fluorescence due to CeO_2 nanoparticles in HUVE cells.

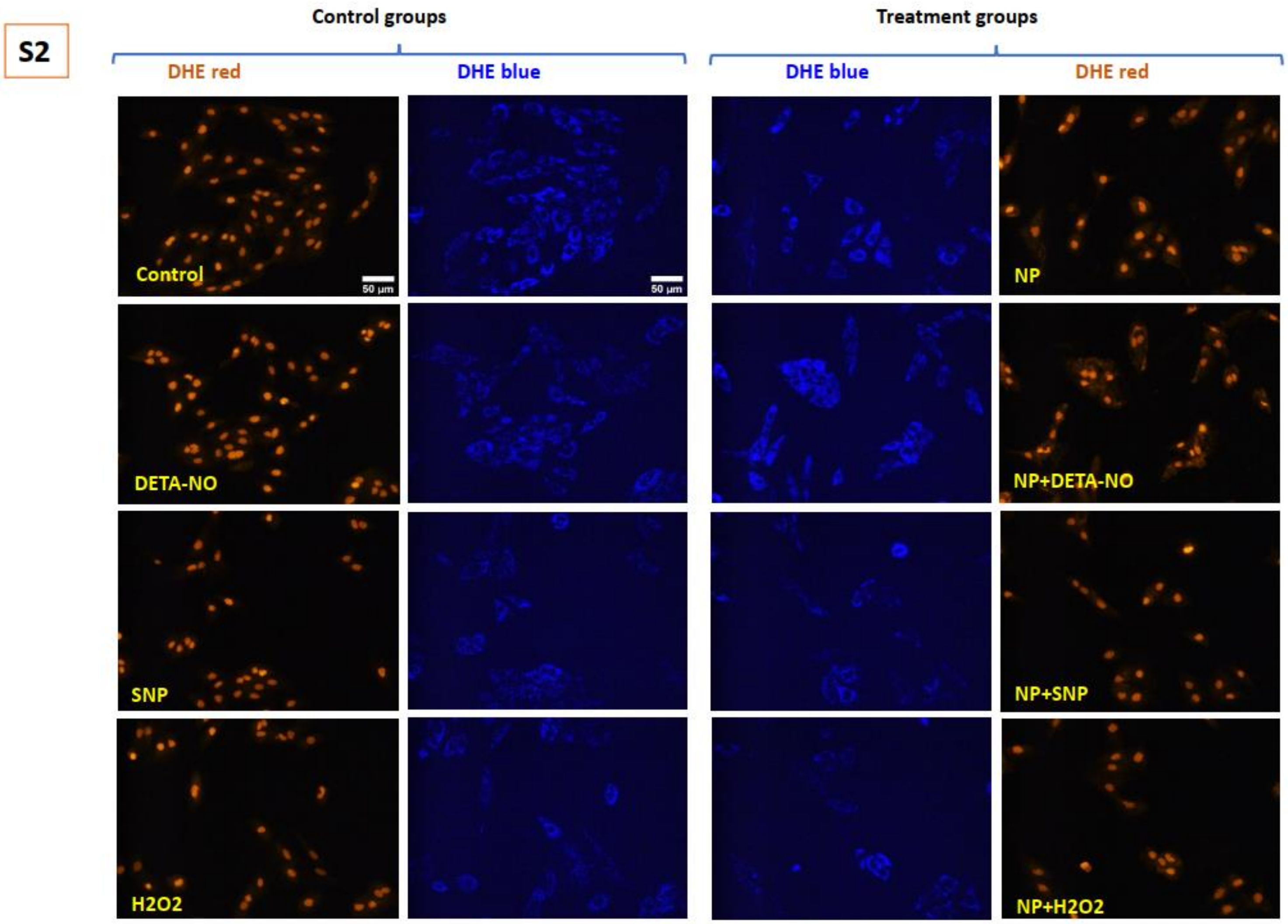

Figure S2: DHE red emission images with their blue emission counterparts.
